# Supplementary material for: Specific excitatory connectivity for feature integration in mouse primary visual cortex
Source: PLoS Comput Biol. 2017 Dec 14;13(12):e1005888. doi: 10.1371/journal.pcbi.1005888 (PMC5746254; doi:10.1371/journal.pcbi.1005888)
Supplement: S1 Fig — a Characterisation of receptive field location using sparse drifting/rotating grating stimuli. Single-trial OGB calcium responses (black); presentation time of optimal stimulus and sub-optimal stimulus indicated (black and grey bars). Right inset: estimated RF location for the same neuron. b Single-trial OGB calcium response to drifting grating stimuli (black); presentation of optimal stimulus orientation indicated above, all stimulus presentation times indicated below. Right inset: calculation of grating response similarity ρg between two neurons. c Single-trial (grey) and trial-averaged OGB calcium response (black) to natural movie stimuli. Vertical lines indicate timing of movie sequence onset. Right inset: calculation of movie response similarity (ρm), using signal correlations over trial-averaged responses from two neurons. d Pairs of neurons with high signal correlations to natural movies (ρm), which predicts a high probability of connection [21], can have similar or dissimilar grating responses. Pairs of neurons with similar orientation preference are not more likely to have high ρm (e) or high signal correlation to flashed natural scenes ρCa (f) than pairs with dissimilar orientation preference. g Connected pairs are slightly more likely to share similar orientation preferences than unconnected pairs [21,24], but nevertheless span almost arbitrary orientation differences (≈20% of pairs with close to orthogonal orientation preference). h In data from functionally characterized neurons with connections reconstructed under electron microscopy [25], connected pairs are more likely to share similar preferred orientations. An excess of connections was present at orientation preference differences of around 30° (p = 0.005, Monte-Carlo test). Dashed lines: 95% bootstrap confidence intervals (CI). d–e: in vivo two-photon calcium imaging; f–g: in vivo calcium imaging coupled with in vitro simultaneous patching to detect connected pairs; data from [24]. h: in vivo calc [file pcbi.1005888.s001.pdf]

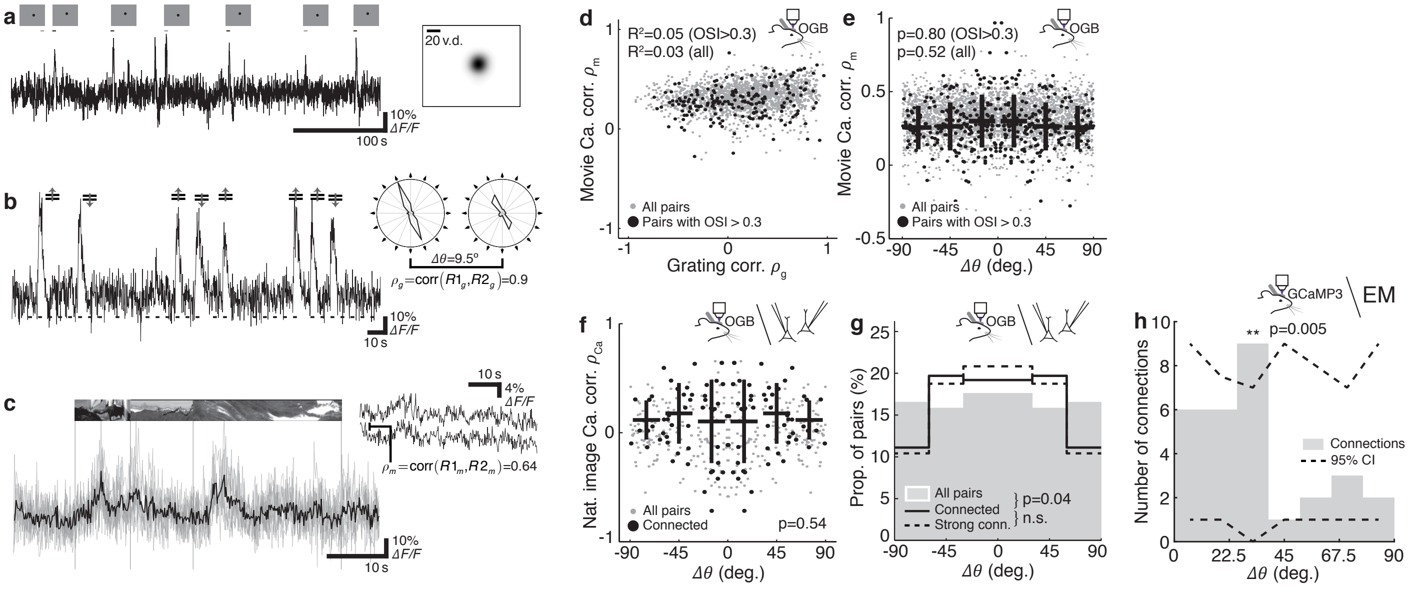

Supporting Figure 1: Connected neurons span a wide range of preferred orientations in mouse V1. a Characterisation of receptive field location using sparse drifting/rotating grating stimuli. Single-trial OGB calcium responses (black); presentation time of optimal stimulus and sub-optimal stimulus indicated (black and grey bars). Right inset: estimated RF location for the same neuron. **b** Single-trial OGB calcium response to drifting grating stimuli (black); presentation of optimal stimulus orientation indicated above, all stimulus presentation times indicated below. Right inset: calculation of grating response similarity ρ_{g} between two neurons. **c** Single-trial (grey) and trial-averaged OGB calcium response (black) to natural movie stimuli. Vertical lines indicate timing of movie sequence onset. Right inset: calculation of movie response similarity (ρ_{m}), using signal correlations over trial-averaged responses from two neurons. **d** Pairs of neurons with high signal correlations to natural movies (ρ_{m}), which predicts a high probability of connection [21], can have similar or dissimilar grating responses. Pairs of neurons with similar orientation preference are not more likely to have high ρ_{m} (**e**) or high signal correlation to flashed natural scenes ρ_{Ca} (**f**) than pairs with dissimilar orientation preference. **g** Connected pairs are slightly more likely to share similar orientation preferences than unconnected pairs [21,24], but nevertheless span almost arbitrary orientation differences (≈20% of pairs with close to orthogonal orientation preference). **h** In data from functionally characterized neurons with connections reconstructed under electron microscopy [25], connected pairs are more likely to share similar preferred orientations. An excess of connections was present at orientation preference differences of around 30° ($p = 0.005$, Monte-Carlo test). Dashed lines: 95% bootstrap confidence intervals (CI). d–e: in vivo two-photon calcium imaging; f–g: in vivo calcium imaging coupled with in vitro simultaneous patching to detect connected pairs; data from [24]. h: in vivo calcium imaging coupled with electron microscopy (EM) reconstruction to identify connected neurons; data from [25]. e–f: Kruskal-Wallis tests; g: Ansari-Bradley test; h: Monte-carlo test. n.s.: $p > 0.05$. Strong connections: strongest 50% of connected pairs, measured by EPSP amplitude. Corr: correlation; conn.: connection.
