# Supplementary material for: Specific excitatory connectivity for feature integration in mouse primary visual cortex
Source: PLoS Comput Biol. 2017 Dec 14;13(12):e1005888. doi: 10.1371/journal.pcbi.1005888 (PMC5746254; doi:10.1371/journal.pcbi.1005888)
Supplement: S2 Fig — a The network stability regimes in the parameter space defined by total inhibitory weight gI∙nI and total excitatory weight gE∙nE for a random network (proportion of specific synapses s = 0%). Nominal parameter estimates for rodent cortex (cross) place the network in a regime that requires inhibitory feedback for stability (an ISN; [50]), but which does not lead to competition between excitatory neurons. Inhibition must be unrealistically strengthened to obtain competition (100× and 200× estimates for rodent cortex; top of panel; shading indicates competition). However, overly-strong inhibition leads to inhibition-driven oscillations (IO). b When the proportion of specific synapses s is raised to 20%, nominal parameters for rodent cortex permit competition (shading indicates strength of competition; see Methods). Note that the maximum excitatory strength permitted while maintaining network stability is reduced. c When s = 40%, nominal parameters for rodent cortex become unstable (cross is just outside stable region). d Network stability regimes for the parameter space defined by s and gE∙nE, with nominal value chosen for gI∙nI (crosses in a–c). Nominal value for gE∙nE is indicated by a dashed line. Both excitatory strength gE∙nE and the proportion of specific synapses s affect network stability and the strength of competition. Abbreviations: gI,E: Synaptic strength per inhibitory or excitatory synapse; nI,E: Number of synapses made by each inhibitory or excitatory neuron; AS: Intrinsically stable network, stable in the absence of inhibition; ISN: Inhibition-Stabilised Network, requiring inhibitory feedback for stability; Exp: Runaway activity due to exponentially divergent unstable fixed point; IO: Oscillatory activity due to strong inhibition. a.u.: arbitrary units. (PDF) [file pcbi.1005888.s002.pdf]

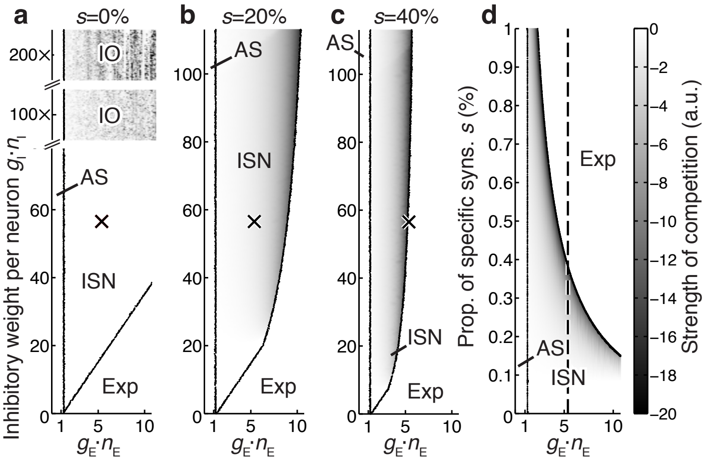

Supporting Figure 2: Estimated parameters for cortex place it in an Inhibition-Stabilised Network (ISN) regime, with competition provided by specific excitatory connectivity.

a The network stability regimes in the parameter space defined by total inhibitory weight $g_I \cdot n_I$ and total excitatory weight $g_E \cdot n_E$ for a random network (proportion of specific synapses $s = 0\%$). Nominal parameter estimates for rodent cortex (cross) place the network in a regime that requires inhibitory feedback for stability (an ISN; [50]), but which does not lead to competition between excitatory neurons. Inhibition must be unrealistically strengthened to obtain competition (100× and 200× estimates for rodent cortex; top of panel; shading indicates competition). However, overly-strong inhibition leads to inhibition-driven oscillations (IO).
b When the proportion of specific synapses *s* is raised to 20%, nominal parameters for rodent cortex permit competition (shading indicates strength of competition). Note that the maximum excitatory strength permitted while maintaining network stability is reduced. **c** When $s = 40\%$, nominal parameters for rodent cortex become unstable (cross is just inside unstable region).
d Network stability regimes for the parameter space defined by *s* and $g_E \cdot n_E$, with nominal value chosen for $g_I \cdot n_I$ (crosses in a–c). Nominal value for $g_E \cdot n_E$ is indicated by a dashed line. Both excitatory strength $g_E \cdot n_E$ and the proportion of specific synapses s affect network stability and the strength of competition. Abbreviations: $g_{I,E}$: Synaptic strength per inhibitory or excitatory synapse; $n_{I,E}$: Number of synapses made by each inhibitory or excitatory neuron; AS: Intrinsically stable network, stable in the absence of inhibition; ISN: Inhibition-Stabilised Network, requiring inhibitory feedback for stability; Exp: Runaway activity due to exponentially divergent unstable fixed point; IO: Oscillatory activity due to strong inhibition.
