# Supplementary material for: Specific excitatory connectivity for feature integration in mouse primary visual cortex
Source: PLoS Comput Biol. 2017 Dec 14;13(12):e1005888. doi: 10.1371/journal.pcbi.1005888 (PMC5746254; doi:10.1371/journal.pcbi.1005888)
Supplement: S3 Fig — a Under the non-specific connectivity model, synapses between pairs of neurons are formed without regard to functional response similarity of the neurons. Neurons form synapses stochastically, according to spatial proximity. Two example pairs of neurons are shown, and their responses to a set of grating and plaid stimuli. b Neurons with similar responses to grating stimuli (high ρg) have similar responses to plaid stimuli (high ρp), and vice versa. Conn.: connectivity; stim.: stimuli. (PDF) [file pcbi.1005888.s003.pdf]

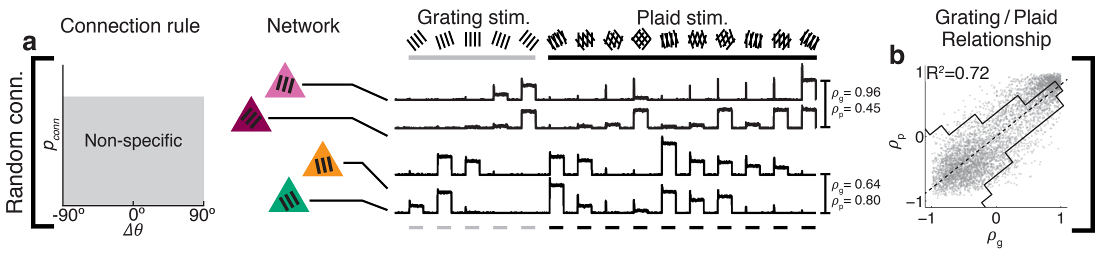


**Supporting Figure 3: Grating and plaid responses are highly correlated in a model with random connectivity. a** Under the non-specific connectivity model, synapses between pairs of neurons are formed without regard to functional response similarity of the neurons. Neurons form synapses stochastically, according to spatial proximity. Two example pairs of neurons are shown, and their responses to a set of grating and plaid stimuli. **b** Neurons with similar responses to grating stimuli (high $\rho_g$) have similar responses to plaid stimuli (high $\rho_p$), and vice versa.
